# Consensus statement of the Italian society of colorectal surgery (SICCR): management and treatment of pilonidal disease

**DOI:** 10.1007/s10151-021-02487-8

**Published:** 2021-06-27

**Authors:** M. Milone, L. Basso, M. Manigrasso, R. Pietroletti, A. Bondurri, M. La Torre, G. Milito, M. Pozzo, D. Segre, R. Perinotti, G. Gallo

**Affiliations:** 1 Department of Clinical Medicine and Surgery, “Federico II” University, Naples, Italy; 2 “Pietro Valdoni” Department of Surgery, Policlinico “Umberto I”, “Sapienza” University of Rome, viale del Policlinico 155, 00161 Rome, Italy; 3 Department of Advanced Biomedical Sciences, “Federico II” University, Naples, Italy; 4 Surgical Coloproctology, “Val Vibrata” Hospital, “L’Aquila” University, Sant’Omero, TE Italy; 5 Department of General Surgery, ASST Fatebenefratelli Sacco, “Luigi Sacco” University Hospital, Milan, Italy; 6 Coloproctology Unit, “S. Anna” Clinic, Pomezia, RM Italy; 7 Department of General Surgery, “Tor Vergata” University, Rome, Italy; 8 Department of General Surgery, “Degli Infermi” Hospital, Biella, Italy; 9 General Surgery Operative Unit, “Città di Bra” Clinic, Bra, CN Italy; 10 Department of Medical and Surgical Sciences, “Magna Graecia” University, Catanzaro, Italy

**Keywords:** Pilonidal sinus, Pilonidal disease PD, Operative management, Minimally invasive approach, Consensus

## Abstract

Pilonidal disease (PD) is a relatively common, benign but challenging condition of the natal cleft. This consensus statement was drawn up by a panel of surgeons, identified by the Italian Society of Colorectal Surgery (SICCR) as having a “special interest” in PD, with the aim of recommending the best therapeutic options according to currently available scientific evidence. A three-step modified-Delphi process was adopted, implying: (1) choice of the panelists; (2) development of a discussion outline and of target issues; and (3) a detailed systematic review of the current literature. The agreement/disagreement level was scored on a five-point Likert scale as follows: “A + : strongly agree; A–: agree; N: unsure/no opinion; D–: disagree; D + : strongly disagree. Each panelist contributed to the production of this manuscript, and the final recommendations were reviewed by the Clinical Practice Guidelines Committee.

## Introduction

Pilonidal disease (PD) is a relatively common, benign but challenging condition, normally, albeit not exclusively [1], involving the natal cleft. PD afflicts around 26 people in 100,000 [2], and its ideal treatment is controversial. In 2015, the Italian Society of Colorectal Surgery (SICCR) issued treatment guidelines [3], inspired by those of the American Society of Colorectal Surgeons [4]. However, the current surgical arena has been recently enriched by relatively new surgical approaches and new evidence. Therefore, the Italian Society of Colorectal Surgery (SICCR) put together an expert consensus statement based on the available literature.

## Methodology

The present consensus statement was drawn up based on the opinion of a panel composed of surgeons identified by the Italian Society of Colorectal Surgery (SICCR) as having a special interest in PD, with the aim of identifying the best therapeutic option(s) to treat PD, according to currently available scientific evidence. The consensus was drawn up according to a modified-Delphi process (5), characterized by three principal steps: (1) choice of panelists, (2) development of a discussion outline and of core issues, and (3) a detailed systematic review of the current literature.

To ensure the inclusion of all available studies, a detailed search for PD was performed in the electronic databases (PubMed, Web of Science, Scopus, EMBASE) with several combinations of keywords: pilonidal sinus, pilonidal sinus disease, etiology, risk factors, diagnosis, surgery, open healing, VAC-therapy, drainage, midline, off-midline, flap, Limberg, Karydakis, minimally invasive treatment, sinusectomy, sinotomy, Gips, endoscopic pilonidal sinus treatment (EPSiT), video-assisted ablation of pilonidal sinus (VAAPS), satisfaction, recurrence, infection, epilation. The literature search included all papers published through July, 2020.

In May, 2020, the invited panelists were asked to agree/disagree with statements based on the current literature, and to submit any comment(s) in case of disagreement. These statements addressed the most important PD related issues, such as: classification, diagnosis, surgical treatment of acute, chronic, and recurrent PD, perioperative management. Each statement was graded according to the criteria adopted by the American College of Chest Physicians, summarized in Table 1 [6], and taking into consideration the relevance of the various levels of evidence, and grades of recommendation**.**

**Table 1:** Grades of recommendation, assessment, development, and evaluation system grading recommendations

	Description	Benefit vs. risk and burdens	Methodological quality of supporting evidence	Implications
1A	Strong recommendation, high-quality evidence	Benefits clearly outweigh risk and burdens or vice versa	RCTs without important limitations or overwhelming evidence from observational studies	Strong recommendation, can apply to most patients in most circumstances without reservation
1B	Strong recommendation, moderate-quality evidence	Benefits clearly outweigh risk and burdens or vice versa	RCTs with important limitations (inconsistent results, methodological flaws, indirect or imprecise) or exceptionally strong evidence from observational studies	Strong recommendation, can apply to most patients in most circumstances without reservation
1C	Strong recommendation, low- or very-low-quality evidence	Benefits clearly outweigh risk and burdens or vice versa	Observational studies or case series	Strong recommendation but may change when higher quality evidence becomes available
2A	Weak recommendation, high-quality evidence	Benefits closely balanced with risks and burdens	RCTs without important limitations or overwhelming evidence from observational studies	Weak recommendation, best action may differ depending on circumstances or patient or societal values
2B	Weak recommendations, moderate-quality evidence	Benefits closely balanced with risks and burdens	RCTs with important limitations (inconsistent results, methodological flaws, indirect or imprecise) or exceptionally strong evidence from observational studies	Weak recommendation, best action may differ depending on circumstances or patients’ or societal values
2C	Weak recommendation, low- or very-low-quality evidence	Uncertainty in the estimates of benefits, risks, and burden; benefits, risks, and burden may be closely balanced	Observational studies or case series	Very weak recommendations; other alternatives may be equally reasonable

*RCTs* Randomized controlled trials

The agreement/disagreement level was scored on a five-point Likert scale as follows: “A + : strongly agree; A–: agree; N: unsure/no opinion; D–: disagree; D + : strongly disagree [7].

Each panelist contributed to the production of this manuscript, and the final recommendations were reviewed by the Clinical Practice Guidelines Committee.

An external reviewer examined the answers provided, and, in case of disagreement, the statements were modified and submitted again to each panelist until complete accordance was reached. Each expert contributed to the production of this manuscript, and the final recommendations were reviewed by the Clinical Practice Guidelines Committee of SICCR.

### Classification

#### No widely used classification of PD has been developed (1C)

Data about classification of PD in the current literature are scarce, and there are no randomized controlled trials evaluating the impact of sinus characteristics on the surgical choice and on surgical outcomes.

A systematic review of the classification systems for PD was performed by Beal et al. [8], considering seven studies [9–15] and featuring several characteristics, including: the presence of a single pit or of multiple pits along the midline, the presence of unilateral or bilateral pits near the midline, the presence of lateral pits within or outside the navicular area as defined by Tezel [13], the recurrence of PD, or the presence of an abscess. Only one classification system recorded the patients’ features as well as characteristics of the disease [14]. Only the location of the sinus was present in all classifications.

**Expert statement: there is no validated classification of PD, even if sinus characteristics may modify surgical decision making and postoperative outcome. Features to be considered should cover: number of pits, their location in relation to midline, distance of the most caudal pits from the anal verge, and the presence of previous incisions or scars. [complete agreement at 2nd round]**

### Diagnosis

#### Diagnosis is based on clinical aspects, physical examination, and disease-specific history (1C)

The diagnosis of PD is mainly clinical, based on signs, symptoms, and physical examination. Patients often complain of severe pain or swelling in the sacrococcygeal area, and, in case of an acute abscess, fever may be an additional symptom.

Physical examination often shows the presence of pits along the natal cleft and/or on the buttocks, far from the midline. Routinely, diagnostic imaging and laboratory tests are not necessary. However, in rare cases, especially in those near the anal verge, it is important to distinguish PD from other perianal conditions such as: cryptogenic perianal fistula, septic anal fissure, gluteal abscess, hidradenitis suppurativa, Crohn’s disease, ulcerative colitis, syphilis, tuberculosis (TB), epidural abscess, other soft tissue infections (folliculitis, furuncles, or carbuncles, etc.), dermoid cyst (teratoma or germ cell tumor). In these cases, a thorough anorectal examination, a proctoscopy, a trans-rectal ultrasonography, or other diagnostic imaging examination should be performed to exclude or to confirm diagnosis of PD [16–18].

**Panel statement: the diagnosis of PD is clinical, although anorectal examination or diagnostic imaging should be performed in case of PD near the anal verge, in order to rule out or to confirm the presence of other anorectal disease(s). [complete agreement at 2nd round].**

### Operative management

#### The mainstay of treatment of an acute pilonidal abscess is simple incision and drainage, regardless of whether it is a primary or recurrent episode. Debridement or primary excision of the pilonidal abscess would be ideal, but depends on the clinical setting. (1B)

Acute pilonidal abscess usually presents with redness, tenderness, pain, and the presence of a fluctuant area in the sacrococcygeal region, sometimes with fever. At this stage, usually all that can be done is simple incision and drainage of the abscess [19]. However, this procedure fails to cure the chronic inflammation due to the foreign body reaction typical of PD, leading to an elusive and temporary healing with re-epithelization of the sinus tract [20]. Therefore, simple incision and drainage carry a recurrence rate up to 42%, which compels these patients to need and seek an additional and, hopefully conclusive, treatment [21].

Several primary treatment options have been described for the conclusive treatment of the pilonidal abscess. First, in a randomized controlled trial (RCT) comparing simple incision and drainage with or without debridement of the sinus tract, debridement was associated with a higher complete healing rate at 10 weeks (96 vs. 79%, *p* = 0.001) and a lower recurrence rate after a follow-up of 65 months (11 vs. 45%, *p* = 0.001) [21].

These results were recently confirmed by a meta-analysis of debridement and laying open, which showed a recurrence pooled rate (DerSimonian and Laird random effects) [22] of 4.47% (95% CI = 0.029–0.063), in both acute and chronic PD [23]. Matter et al. [24] compared drainage alone with primary excision of a pilonidal abscess. The recurrence rate was 55 and 41%, respectively. In a comparison between drainage followed after 3 weeks by excision and primary closure with excision and secondary healing, Hosseini et al. [25] demonstrated a higher recurrence rate of the abscess (14 vs. 0%, *p* < 0.05) within 12 months after delayed excision and primary closure.

Recently, minimally invasive techniques have been suggested to also treat the acute presentation of PD [26, 27]. For instance, in a small study comparing simple incision and drainage vs. endoscopic pilonidal abscess treatment (EPAT) (20 patients in each group), EPAT seemed to be associated with a shorter time to wound healing (16 vs. 35 days, *p* = 0.0018), but the same number of cases required further conclusive surgery (20% [26] or 21% [27]).

**Panel statement: the standard treatment for acute pilonidal abscess remains simple incision and drainage. Debridement or primary excision of the pilonidal abscess or minimally invasive techniques could, also, be a valid alternative in the individual patient. [complete agreement at 2nd round].**

#### The most appropriate surgical treatment of chronic PD sinus is controversial (1B)

At least one paper has shown the benefits of open healing over primary closure after the excision of chronic PD in terms of recurrence [28]. On the other hand, the recurrence rate was significantly higher in patients left open compared to patients after Limberg flap (7/15 vs. 1/24 *p* = 0.005) [29], while another research showed a substantially overlapping recurrence rate after Z-plasty and open wound [30].

When comparing “midline” with “off-midline” primary closure, the current literature advocates “off-midline” suturing, at least in terms of surgical site infections (SSI), and recurrence rates [31–33].

The introduction of minimally invasive, “targeted” procedures has significantly changed the surgical approach to chronic PD [34–43]. These techniques (Table 2) were first launched in the ‘60 s, and became popular in North America in the 1980s, after Bascom proved that PD is a skin condition, thus providing solid grounds for a “targeted” and minimally invasive surgical approach [35, 36]. Recently, a number of minimally invasive approaches have become popular, parallel to their feasibility and to expectations of both patients and of private or public health services, always trying to minimize costs and favoring less dressings, faster recovery, and prompt return to active work or to school/university activities.

**Table 2:** Minimally invasive surgery techniques to treat pilonidal disease

	Description	Year	Reference
1	Lord-Millar	1965	[34]
2	Bascom’s pit-picking	1980	[35]
3	Sinusectomy	2002	[38]
4	Sinotomy	2005	[110]
5	Punch biopsy	2008	[40]
6	Video-assisted ablation of pilonidal sinus (VAAPS)	2014	[42]
7	Endoscopic pilonidal sinus treatment (EPSiT),	2014	[41]

However, until now, few RCTs have compared these minimally invasive techniques vs. time-honored standard surgical treatments [44–46].

Recently, Popeskou et al. [44], while comparing sinusectomy with off-midline primary closure, had to prematurely interrupt their trial because of the longer wound healing time after sinusectomy, in contrast with the expected results.

While comparing both short- and long-term outcomes of video-assisted ablation of pilonidal sinus (VAAPS) vs. Bascom “cleft lift”, Milone et al. [45, 46] demonstrated that VAAPS was associated with shorter time off work and lower postoperative infection rates, less pain, and higher patient satisfaction, with a comparable 5-year recurrence rate.

Finally, a study compared sinusectomy and endoscopic treatment [47], showing that endoscopy was associated with a recurrence rate lower than sinusectomy, but overlapping postoperative pain and patient satisfaction. However, recurrences after sinusectomy were unacceptably high (25%).

**Panel statement: validated operative techniques for the treatment of chronic PD include: open healing, off-midline primary closure, and minimally invasive techniques. [complete agreement at 2nd round].**

#### In the case of primary closure, off-midline closure should be the treatment of choice. Employment of drains should be tailored to the individual patient (1B)

Six studies compared surgical midline against off‐midline closure. Healing times were faster after off- midline closure (MD 5.4 days, 95% CI 2.3–8.5). SSI rates were higher after midline closure (RR 3.72, 95% CI 1.86–7.42) and recurrence rates were higher after midline closure (Peto OR 4.54, 95% CI 2.30–8.96) [48].

A meta-analysis of RCTs, comparing different techniques with primary closure for chronic PD, showed that open radical excision and primary midline closure should be abandoned and that sinusectomy/sinotomy or “en bloc” resection with off midline primary closure should be the preferred approaches [49].

In a long-term analysis, the recurrence rate after open healing, midline closure and off-midline closure was 17.9%, 16.8%, and 10%, respectively. Unfortunately, statistical analysis was not performed, and this study only focused on recurrence [50].

**Panel statement: in the case of primary closure, off-midline closure must be considered to be the gold standard because it is associated with better postoperative outcomes compared to midline closure. [complete agreement at 1st round].**

Various “off-midline” techniques have been described over the years. The Karydakis flap (K-flap) allows to perform an “off-midline” suture employing a mobilized fasciocutaneous flap secured to the sacrococcygeal fascia. In a survey on 7471 patients treated from 1966 through 1990 and with a follow-up from 2 to 20 years, Karydakis [51] showed a recurrence rate of 1.0% and a wound complication rate of < 8%. In a recent RCT comparing K-flap with open healing [52], K-flap was associated with a significantly lower time to wound healing, return to work, wound complications, and recurrence.

In the Limberg (rhomboid) flap technique, all sinuses are excised and a rotating lipocutaneous flap is used to lower down the natal cleft [53]. A few RCTs [54–57] and meta-analyses of RCTs [58–60] have demonstrated that Karydakis and Limberg flaps have similar good outcomes in terms of post-operative SSI, return to work, wound healing, and recurrence rate, although the K-flap seems to be related to a higher occurrence of seroma.

The “cleft lift” technique was first described by Bascom in 1987 [61–63], and, following pilonidal excision, is based on the employment of an asymmetrical skin flap to cover deep natal clefts, resulting in a suture off the midline. Initially the “cleft lift” was employed in refractory or unhealed PD, but several cases have shown that it has a role in the primary approach [64–66]. Only one RCT comparing Bascom cleft lift and Limberg flap has been published in the last decade, showing that, although both techniques achieved good results during the early period, the Bascom cleft lift provides shorter operative times and better quality of life during the early postoperative period [67].

Several other flap techniques have been developed over the years, such as the V–Z advancement flap, Z-plasty, and parasacral perforator flap techniques. These flap techniques have been successfully employed in the treatment of complex PD, with a complete healing rate > 90%, as reported in a few series [68–70].

In a randomized trial comparing Z-plasty and delayed healing by secondary intention, Fazeli et al. [30] showed that Z-plasty was associated with a shorter time to both wound healing and return to normal daily activities, while there were no significant differences in terms of bleeding, hematoma, infection, and recurrence rate.

Despite these results, it is important to emphasize that these techniques may require general anesthesia, prolonged hospitalization, and surgeons dedicated to these procedures [71].

**Panel statement: it is not possible to identify the best off-midline technique, all being validated procedures. [complete agreement at 2nd round].**

Several studies have assessed the use of drains after primary closure [72–75]. The results of a nonrandomized trial have indicated that the employment of drains after pilonidal excision and primary closure was associated with a lower rate of wound healing, without differences in the recurrence rate [72].

In a randomized comparison between employment of drains vs. no-drains after primary closure, Milone et al. [73] demonstrated that drains did not achieve a faster wound healing, and, on the contrary, they were associated with lower patient tolerance. When adopted after flap techniques, drains are associated with a lower incidence of fluid collections but no actual difference in wound infection [74], while Erdem et al. showed that Limberg flaps with no drains in place result in shorter hospital stays without deleteriously affecting the surgical results of wide excision and primary closure with well-vascularized tissue [75]. A relatively recent meta-analysis [76] of randomized trials in patients undergoing either Karydakis or Limberg flap tried to identify the association between placement of a drain and the infection and recurrence rates, suggesting that, despite a trend toward a reduction in wound infection and recurrence rates, drains were not associated with overall better outcomes.

**Panel statement: when a primary closure is performed, the employment of drains depends on the surgeon’s preference and on the individual patient. [complete agreement at 2nd round].**

#### The benefits of open healing vs. primary closure are controversial (1B)

Traditional surgery for chronic PD can be divided in two categories: excision with primary closure (including midline and off-midline sutures and reconstruction with flaps) and excision with healing by secondary intention (open healing) [50, 77].

The 2010 Cochrane Systematic Review [48], compared open healing with primary closure after excision of PD, including 17 RCTs published from 1987 through 2009. The results suggested that open healing was associated with a longer time to wound healing over primary closure (range: 41–91 days vs. 10–27 days). The SSI rate was similar in the two groups. On the contrary, open healing was associated with a recurrence rate significantly lower than primary surgical closure, with a reduction of the recurrence risk of 35%. When assessing time off daily activities, a significant clinical improvement was found in the primary closure group vs. open healing.

A recent a meta-analysis of 5 RCTs compared flap vs. lay open excision [78]. The results showed a non-significant trend toward less recurrences in case of flaps vs. the laying open technique. Time to complete wound healing and time off work were significantly shorter after the flap technique, while the SSI rate was similar within both groups.

In 2014, Enriquez-Navascues et al. [49] performed a meta-analysis of 25 RCTs comparing the results of different open healing and primary closure approaches after excision of PD. In studies comparing sinusectomy/sinotomy versus open “en bloc” resection, no significant differences were found in terms of time to healing and recurrence rate between the two groups, while return to daily activities was faster in the sinusectomy group.

**Panel statement: open healing should be limited to complex cases, since the benefits on recurrence are not clear and the postoperative recovery may be longer. [complete agreement at 2nd round].**

Open healing, aside from causing patient discomfort, may require frequent painful dressings, and close clinical observation. Dressings should provide an optimal environment for wound healing, and the so called “advanced dressings” do this by simple physical or chemical means, typically by controlling moisture levels, with the aim of optimizing wound cleansing and re-epithelization (for example, calcium alginate, film, foam, hydrocolloid, and hydrogel dressings) [79–82].

In a recent RCT comparing three different dressing methods after pilonidal surgery [83], dressings with hydrogel or alginate and hydrocolloid compounds reduced the average number of days off daily activities in comparison with Vaseline gauze (modified method dressing) and sterile gauze (standard method). However, so far, no single dressing method has been scientifically proven to be superior to others [84].

A RCT was conducted by Mohammadi et al. to test the effect of platelet-rich plasma (PRP) on wound healing after sinus surgery and showed that PRP was associated with a significantly faster healing process and return to routine daily activities compared to classic wound dressing with sterile gauze [85].

Negative pressure wound therapy (NPWT) was also introduced with the purpose of speeding up the process of wound healing. In a RCT comparing NPWT and standard dressings after open healing, Biter et al. [86] showed that NPWT was associated with faster wound healing within the first two weeks after surgery. However, no significantly shorter time to wound healing and return to daily activities was related to the use of NPWT. On the contrary, NPWT required a longer hospital stay.

Panel statement: in case of wide pilonidal excision and open healing, NPWT is recommended. Advanced dressings could be a valid alternative. [complete agreement at 2nd round]

#### Minimally invasive techniques may be safe and effective in the treatment of chronic pilonidal sinus (1B)

A minimally invasive approach was first described by Lord-Millar in the 1960s [34]. Later, in the 1980s, Bascom developed the concept of “targeted procedure”, aimed at treating and removing only the diseased tissues, leaving alone any unaffected and healthy surrounding tissue. This was based on his solid studies on the origin of PD [35–37]. Later, Oncel, Soll and Gips, while adopting the same principles, slightly modified these “targeted procedures” [38–40].

Oncel, in 2002, first reported sinusectomy to treat limited, chronic PD in a series of 40 patients, with a shorter operation time, hospital stay and period off work than excision and marsupialization [38]. Later, Soll, in 2008, described a limited excision procedure, consisting of selective resection of the sinus by means of scalpel or scissors, with a recurrence rate of 5% after a median follow-up of 2 years, and a median time off work of 2 weeks, and of 5 weeks to wound healing [39]. Gips’ “targeted procedure” is also based on the same sound principles of Bascom, and conveniently employs trephines or disposable biopsy punches of various diameters instead of a small scalpel to excise the pits and debride the sinus cavity [40]. In his first analysis on 1,358 patients, Gips reported a recurrence rate of 6.5, 13.2, and 16.2% at 1-, 5-, and 10-year follow-up, respectively. Despite the high recurrence rate, the strength of this technique lies in its repeatability (85% of the patients cured by one procedure, 95% by a second). All these minimally invasive techniques require solid experience and expertise in order not to leave behind any untreated PD.

The meta-analysis by Enriquez-Navascues et al. [48] found four papers [87–90] comparing open limited excision (sinusectomy) or unroofing (sinotomy); although recurrence rate did not differ, all other outcomes favored the limited approach.

More recently, in a RCT comparing conservative sinusectomy and excision with primary “off-midline” closure, Popeskou et al. [44] prematurely stopped their study because of adverse outcomes in the sinusectomy group, since these patients were associated to slower wound healing after three weeks, compared to primary closure.

In 2014, Milone et al. [42] and Meinero et al. [41] independently proposed an endoscopic approach to chronic PD (VAAPS and endoscopic pilonidal sinus treatment [EPSiT], respectively). Both techniques, while merging the now long-lasting principles of “targeted procedure” with technology, seem to be associated with a faster postoperative recovery and wound healing, quicker return to normal daily activities, and higher patient satisfaction when compared to traditional surgical techniques [46].

In a randomized comparison between VAAPS and Bascom cleft lift [45], VAAPS implied a shorter time off work and lower postoperative infection rate, less pain, and higher patient satisfaction. Although these results are encouraging, less is known about the long-term recurrence rate after treatment using endoscopic techniques. Giarratano et al. [91], in a long-term prospective assessment, achieved a recurrence rate of 7.8% with a median follow-up of 25 months.

Another long-term randomized study assessed the 5-year recurrence rate, confirming that this was similar in both groups (endoscopic vs. conventional Bascom cleft lift procedure) [45]. Different results were obtained by Romanszyn et al. [92] in a nonrandomized research comparing endoscopic treatment and Limberg flap to treat complicated PD. The endoscopic procedure had a significantly lower success rate than the Limberg flap procedure, but a lower risk of postoperative complications.

Recently, several systematic reviews and meta-analyses confirmed the feasibility and safety of the endoscopic techniques, associated with a low recurrence rate, a good complete healing rate, and good patient satisfaction [93, 94]. In any case, further investigations are needed.

**Panel statement: minimally invasive treatments are validated techniques that should be the treatment of choice in case of limited pilonidal disease (single pit or multiple pits on the midline). [complete agreement at 2nd round].**

### Management of recurrent pilonidal disease

#### The management of recurrent pilonidal sinus is similar to “de novo” presentation (1C)

Recurrence can be defined as the additional outbreak of signs and symptoms of PD after a disease-free interval following complete wound healing. Risk factors for recurrence are adolescence, sinus number, cavity diameter, and primary closure [95, 96].

The surgical treatment of patients with recurrent disease does not differ from the surgical treatment of primary PD. In case of a recurrence with an abscess, incision and drainage prevail, while in case of chronic recurrent PD, a flap based procedure may be indicated following sinus excision with scarring.

In a randomized evaluation of recurrent PD comparing Limberg flap and modified asymmetric Limberg flap, Cihan et al. [97] demonstrated that a modified asymmetric Limberg flap was associated with a lower infection rate, shorter hospital stay, and shorter time off work, with a trend toward less recurrence after modified asymmetric Limberg flap.

In 2019, Meinero et al. [98] completed a multicenter prospective study on 122 patients with recurrent PD treated with EPSiT, showing a complete healing rate of 95% and a recurrence rate of 5.1%, while time off work was 3 days. Similar results were also obtained by Manigrasso et al. [99] in a retrospective analysis of 63 patients, with a recurrence rate of 4.7%, 11.7%, and 23% after 1, 3, and 5 years, respectively. The rate of incomplete wound healing was 4.7%, and time off work was 3.5 days.

Although these results encourage a minimally invasive approach to treat recurrent PD, data in the current literature are still limited.

**Panel statement: surgical procedures for recurrent PD do not differ from those of primary PD. Even in the case of recurrence, proper surgical treatment should be tailored to the individual patient. [complete agreement at 2nd round].**

### Perioperative management

#### Antibiotics have a limited role in wound infections and recurrence (1B)

Antibiotics could be useful in three different settings: perioperative prophylaxis, postoperative care, and local treatment. In relation to perioperative prophylaxis, Sondeena et al. [100], in a randomized comparison of a single preoperative dose of 2 g intravenous (i.v.) cefoxitin vs no cefoxitin, did not demonstrate statistically significant differences in terms of infection and healing rates.

Similar results were obtained by Kundes et al. [101], who compared the preoperative use and the non-use of a single dose of 1 g (i.v.) cefazolin sodium plus metronidazole 500 mg i.v., within 60 min before skin incision. Even in this study, the use of prophylactic antibiotics did not significantly impact on SSI and recurrence rates.

On the contrary, data in the literature about the use of antibiotics postoperatively are controversial. In a randomized, double-blinded pilot study, Chaudhuri et al. [102] compared wound infections after excision of pilonidal sinuses and primary closure using either a single pre-operative dose of (i.v.) metronidazole, or both (i.v.) cefuroxime and (i.v.) metronidazole preoperatively, and co-amoxiclav 375 mg orally every 8 h, for 5 postoperative days). The single-drug group was associated with a significantly higher wound infection rate after 2 (*p* < 0.0001) and 4 weeks (*p* = 0.03).

In a RCT [103], the adoption of clindamycin as an adjunct to excision and primary closure translated into a shorter wound healing time both in case of excision and primary closure, and of excision alone. On the other hand, a recent review of 7 studies, totalling 690 patients, showed no advantage when single-dose prophylaxis was compared with no prophylaxis or to a long course of antibiotics in several single- and double-coverage antibiotic regimens [104].

In relation to the efficiency of local antibiotics, data are limited and conflicting. In a randomized trial, Yetim et al. [105] compared the use of oral postoperative antibiotics for 7 days and the placement of gentamicin-absorbed collagen sponges after pilonidal sinus excision and primary closure. The use of gentamicin-absorbed collagen sponges was associated to a lower infection and recurrence rate, and a shorter hospital stay. Similar results were obtained when comparing primary closure and gentamicin sponge vs. secondary healing [106]. On the contrary, a randomized comparative study on primary closure with or without gentamicin-collagen sponges showed no differences between the two treatments [107].

Recently, a review and meta-analysis of the use of collagen showed less wound infections, but no significant influence on wound healing or disease recurrence [108].

Panel statement: pre- and postoperative antibiotic use do not significantly impact wound infection and dehiscence rates

#### Hair removal from the natal cleft may be useful as an additional treatment after excision of the pilonidal sinus (1C)

The rationale for hair removal lies in the eradication of risk factors for the development of PD. Light amplification by stimulated emission of radiation (LASER) epilation may be useful as an additional treatment after excisional surgery. Several studies have demonstrated a lower postoperative recurrence rate in case of hair removal, after complete wound healing [109, 110]. On the contrary, the employment of a razor (shaving) in the immediate postoperative period seemed to be associated with a higher recurrence rate [111].

However, data about the real advantages of hair removal as an adjunct to excisional surgery are controversial. A prospective RCT, comparing pre- and postoperative LASER hair removal with no hair removal, failed to demonstrate that LASER hair removal reduced the recurrence rate [112]. However, in this RCT, LASER epilation was associated with less postoperative pain and higher patient satisfaction than surgery alone.

On the other hand, a randomized comparison between LASER epilation and hair removal by means of a razor or depilatory creams, demonstrated a lower recurrence rate if LASER was used [113]. Recently, the results of a systematic review of the literature on postoperative hair removal showed that postoperative LASER epilation was associated with a lower recurrence rate than razor/cream depilation. The recurrence rate of PD was 9.3% (34 out of 366 patients) in patients who had LASER hair removal, 23.4% (36 out of 154 patients) in those who had razor shaving/cream depilation, and 19.7% (85 out of 431 patients) in those who had no hair removal after surgery [114]. Nevertheless, current evidence of the efficacy of hair removal after pilonidal surgery is still low and needs additional studies.

**Panel statement: in hirsute patients, postoperative epilation is recommended. [complete agreement at 2nd round]**

## Conclusions

The validated surgical techniques for the management of PD are open healing, off-midline primary closure, and minimally invasive “targeted” techniques (sinusectomy and endoscopic approaches).

Surgical management should be individualized and tailored according to the individual PD.

A key challenge for the future will be to develop and validate a classification of PD, which should then assist and guide the surgeon in the management of this disease. Although many surgical aspects of the treatment of PD remain controversial, the panelists recommend the adoption of minimally invasive surgery in cases of limited PD (single/multiple pits on the midline), and traditional open healing for complex cases.
